# Protective Effect of 17β-Estradiol Upon Hippocampal Spine Density and Cognitive Function in an Animal Model of Vascular Dementia

**DOI:** 10.1038/srep42660

**Published:** 2017-02-16

**Authors:** Ying Zhu, Quanguang Zhang, Wenli Zhang, Ning Li, Yongxin Dai, Jingyi Tu, Fang Yang, Darrell W. Brann, Ruimin Wang

**Affiliations:** 1 Neurobiology Institute of Medical Research Center, International Science & Technology Cooperation Base of Geriatric Medicine, North China University of Science and Technology, Tangshan 063000, China; 2 Department of Neuroscience and Regenerative Medicine, Medical College of Georgia, Augusta University, Augusta 30912, USA

## Abstract

The current study examined whether the steroid hormone, 17β-estradiol (E2) can exert long-lasting beneficial effects upon axonal health, synaptic plasticity, dementia-related amyloid-beta (Aβ) protein expression, and hippocampal-dependent cognitive function in an animal model of chronic cerebral hypoperfusion and vascular dementia (VaD). Chronic cerebral hypoperfusion and VaD was induced by bilateral common carotid artery occlusion (BCCAO) in adult male Sprague Dawley rats. Low dose E2 administered for the first 3-months after BCCAO exerted long-lasting beneficial effects, including significant neuroprotection of hippocampal CA1 neurons and preservation of hippocampal-dependent cognitive function when examined at 6-months after BCCAO. E2 treatment also prevented BCCAO-induced damage to hippocampal myelin sheaths and oligodendrocytes, enhanced expression of the synaptic proteins synaptophysin and PSD95 in the hippocampus, and prevented BCCAO-induced loss of total and mushroom dendritic spines in the hippocampal CA1 region. Furthermore, E2-treatment also reduced BCCAO induction of dementia-related proteins expression such as p-tau (PHF1), total ubiquitin, and Aβ1-42, when examined at 6 m after BCCAO. Taken as a whole, the results suggest that low-dose E2 replacement might be a potentially promising therapeutic modality to attenuate or block negative neurological consequences of chronic cerebral hypoperfusion and VaD.

Chronic cerebral hypoperfusion has been implicated as a potentially important pathological factor in mild cognitive impairment, Alzheimer’s disease (AD) and vascular dementia (VaD)^1^,^2^,^3^,^4^. Dementia is a syndrome associated with progressive declines in cognitive capacities and impairments that interfere with daily functioning^5^. Increasing evidence shows that AD and VaD account for most dementia cases, especially in the aging population^6^,^7^. VaD is known to occur when the blood supply to the brain is reduced or inhibited by an impaired vascular system^8^. Accumulation of insoluble amyloid beta (Aβ) in the brain has been proposed as a major factor contributing to the cognitive impairment observed in AD patients^9^. By mimicking such a pathological condition, various animal models have been developed to explore the underlying mechanism of cognitive impairment in VaD. Permanent occlusion of the bilateral common carotid artery (BCCAO) is a well-established method in rats that is used to investigate the effect of chronic cerebral hypoperfusion on cognitive dysfunction with significant injury to the white matter and hippocampal neuronal damage^4^. As such, BCCAO in rats has become a widely used model of VaD over recent years^10^,^11^,^12^,^13^.

17β-estradiol (E2) is a steroid hormone produced from androgens in men and women through the action of the biosynthetic enzyme, aromatase^14^,^15^,^16^,^17^. In females, the ovary is the major E2 generating organ, whereas in males, which have lower levels of circulating E2, adipose tissue is a major site of E2 generation. Following its production, E2 is released into the bloodstream and acts upon various tissues in the body, including the brain, to regulate their function. Basic science and clinical observation studies have provided evidence of a neuroprotective effect of E2 in neurodegenerative diseases such as stroke and AD^18^,^19^,^20^,^21^. There is growing recognition that chronic cerebral hypoperfusion such as can occur in diabetes and vascular disorders may be a critical prodrome to neurodegenerative disorders such as AD and VaD^1^,^2^,^3^,^4^. Chronic cerebral hypoperfusion can lead to decreased neuronal health, neuroinflammation, and increased susceptibility to stressors, which have been implicated to contribute to the pathogenesis of AD and VaD^1^,^3^. While E2 has been shown to be neuroprotective and regulate synaptic plasticity and cognitive function in acute ischemia models^22^,^23^,^24^,^25^,^26^,^27^, it is unknown whether it can prevent the negative neural consequences from chronic cerebral hypoperfusion. Therefore, the goal of the current study was to examine the neuroprotective, as well as the synaptic- and cognitive-preserving effects of chronic E2 in the BCCAO animal model of chronic cerebral hypoperfusion and VaD.

## Results

### 17β-Estradiol preserves spatial memory at 3 months BCCAO

**Figure 1:**
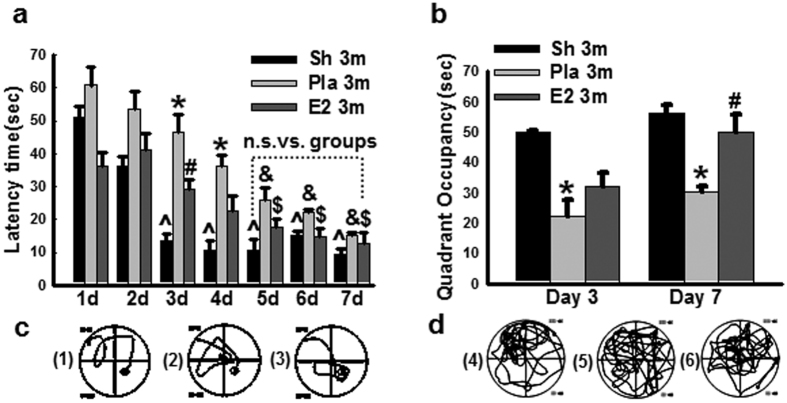
Effect of 17β-estradiol (E2) treatment on cognitive function after BCCAO 3 months. (**a**) Latency trail in Morris water maze, *P < 0.05 vs. sham (Sh) 3-months (3 m) group at the same time-point; ^#^*P* < 0.05 vs. Placebo (Pla) group at the same time-point; ^^^*P* < 0.05 vs. sham group at day one; ^$^*P* < 0.05 vs. E2-treatment group at day one; ^&^*P* < 0.05 vs. Pla group at day one. (**b**) Probe trail in Morris water maze. *P < 0.05 vs. sham and ^#^*P* < 0.05 vs. Pla group at the same time-point. Representative tracks for latency trail (**c**: 1, sham; 2, Pla; 3, E2-treatment) and for probe trail (**d**: 4, sham; 5, Pla; 6, E2-treatment); n = 6/group.

In order to address the potential protective role of E2 in male animals, we first measured circulating E2 levels in the various groups to demonstrate the levels produced by placement of the E2 mini-osmotic pumps. The results revealed that exogenous E2 replacement produced serum E2 levels of 25–33 pg/ml in the male rats at 3 and 6 months after BCCAO (Sup. Fig. 1), which is slightly, but significantly higher than E2 levels in the non-E2 treated Sham and Pla male animal control groups (19–21 pg/ml). We next examined the effect of 3 months of bilateral common carotid artery occlusion (BCCAO 3 m), as well as continuously low dose E2 replacement upon cognitive function of adult male rats using the Morris water maze (MWM) test. As shown in Fig. 1a, on days 1, 2, 3, 4 of MWM testing, BCCAO 3 m + Pla (Pla 3 m) rats displayed a significant increase in latency to find the hidden platform, as compared to sham and E2 3 m rats. However, by days 5, 6, 7, there was no significant difference among any of the groups with regards to latency to find the hidden platform in the MWM. Thus, after 7 days of training, all animals displayed good learning capacity, as evidenced by a significant short latency to find the platform on the last day (day 7) of the trial as compared to that on the first day. On day 3 and day 7 (6 hr following the latency trial), a probe trial was conducted to measure spatial bias for the previous platform location, indicating memory ability of the animals. As shown in Fig. 1b, Pla 3 m rats exhibited a significant decrease in time spent in the platform quadrant, as compared to the sham 3 m control group. Furthermore, E2 treatment significantly preserved spatial memory on day 7 of the probe trial, as compared to the BCCAO 3 m group. Representative tracings illustrating sample paths of rats from the various groups are shown in Fig. 1c,d, respectively.

### Hippocampal CA1 neurons exhibit damage but no cell death at 3 months BCCAO

**Figure 2:**
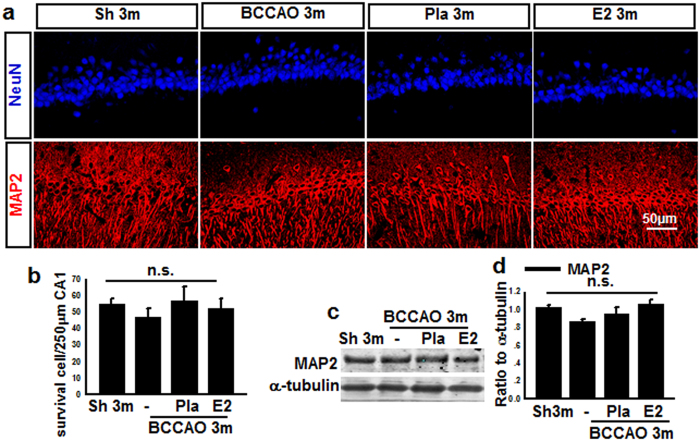
Effects of E2-treatment upon neuronal markers (NeuN, MAP2) in hippocampal CA1 region at 3 months after BCCAO. **(a)** Sections from sham (Sh) 3 m, BCCAO 3 m, Placebo (Pla) 3 m, and E2-treatment 3 m were subjected to immunofluorescent staining for NeuN (Blue), and MAP2 (Red). **(b)** Quantify for NeuN-positive cells in 250 μm length of the medial CA1 pyramidal cell layer. n = 15. **(c, d)** Western blot showed that MAP2 protein expression had no significant change in all groups. n.s. means no significant change between the four groups; n = 5–6, scale bar is 50 μm; magnification is 40×.

It is well known that spatial cognitive ability is a hippocampal dependent function and involves pyramidal neurons in the hippocampal CA1 region^28^,^29^. Since BCCAO 3 m caused a loss of spatial cognitive function, we examined whether this effect could have been due to pyramidal neuronal cell loss in the CA1 region. To examine neuronal cell loss in the CA1 region, we performed immunohistochemistry on hippocampal sections for NeuN, a neuronal marker. Representative photomicrographs of NeuN (blue) immunostaining for all groups are shown in Fig. 2a, while quantification of results from all animals in each group is presented in Fig. 2b (NeuN, surviving cells). As shown in Fig. 2a & b, there was no significant difference in the number of surviving neuron in the hippocampal CA1 region among the different groups. To further confirm the results, expression of the neuronal cytoskeletal protein MAP2 in the CA1 region was examined. As shown in Fig. 2a & c, Western blot and immunohistochemical analysis revealed no significant difference in MAP2 protein expression in the hippocampal CA1 region among the different groups. While the hippocampal CA1 pyramidal neurons may not have increased cell death, they could still have structural and organelle damage following BCCAO that could explain the decreased spatial cognitive performance in BCCAO animals. Thus, we next examined ultrastructural properties of CA1 pyramidal neurons using electron microscopy (EM). EM ultrastructural analysis revealed that CA1 neurons in the BCCAO 3 m group displayed cytoplasm degeneration, dilated rough endoplasmic reticulum and swollen mitochondria with severe broken cristae (Sup. Fig. 2a), although the nuclei showed a regular contour and an unaltered pattern of chromatin distribution. Interestingly, E2 treatment significantly preserved CA1 neuron ultrastructure, which is in agreement with its preservation of spatial cognitive function. Finally, analysis of early-stage apoptosis in the hippocampal CA1 region was performed using immunofluorescent staining for Annexin-V and NeuN. As shown in Sup. Fig. 2b & c, the Annexin-V level in the BCCAO 3 m group was significantly increased compared with sham 3 m control, and E2 replacement markedly reversed the changes, although the number of NeuN-positive cells had no significant change in all groups.

### 17β-Estradiol prevents axonal damage induced by BCCAO 3 months

**Figure 3:**
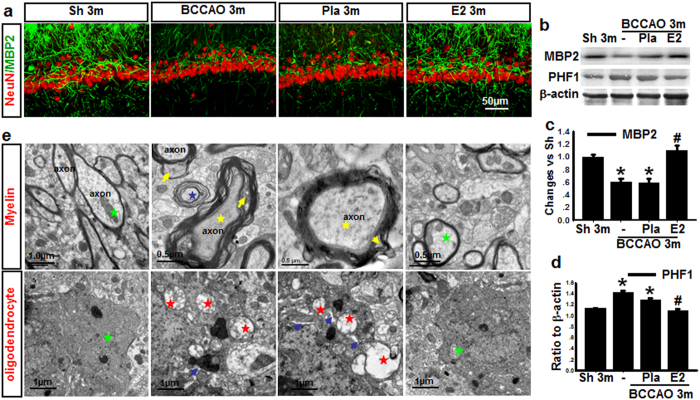
Effects of E2-treatment upon the ultrastructure of axons, myelin, oligodendrocytes, and expression of axonal proteins in the hippocampal CA1 region after chronic BCCAO. (**a**) Representative photographs of double-immunofluorescent staining for myelin basic protein-2 (MBP2) (green) and NeuN (red, neuron marker) in Sham (Sh) 3 m, BCCAO 3 m, Placebo (Pla) 3 m, and E2-treatment 3 m (scale bars 50 μm, magnification 40×). (**b**,**c**,**d**) Western blot analysis for MBP2 and PHF1 protein expression in the hippocampal CA1 region of various groups at BCCAO 3 m. *P < 0.05 vs. sham group, ^#^*P* < 0.05 vs. Pla 3 m group, n = 5–6; (**e**) Ultrastructure of axon, myelin and oligodendrocytes in the hippocampal CA1 region of various groups at BCCAO 3 m. Healthy axon with intact myelin (green star); damaged axon with severe disorganization (yellow star), dense degradation (yellow arrow) and swollen (blue star) myelin sheaths; Healthy oligodendrocytes with intact mitochondria (green star), and damaged oligodendrocytes with dilatation of rough endoplasmic reticulum (blue arrow), swollen mitochondria with broken cristae (red star).

Neuronal degeneration and pathological accumulation of tau are well known as a primary cause leading to memory loss in early dementia^28^,^30^. One of tau’s main functions is to modulate the stability of axonal microtubules. If tau in oligodendrocytes is unable to bind to microtubules because of its hyperphosphorylation, it will result in myelin abnormality^31^,^32^. We thus examined for axonal changes after 3 m BCCAO by examining protein expression of phospho-Tau (tau phosphorylation at Ser^396^/Ser^404^) using a PHF1 antibody, and an antibody to MBP2, a marker for oligodendrocytes in the hippocampal CA1 region following BCCAO with and without E2 treatment. As shown in Fig. 3a, representative double immunofluorescence staining for MBP2 (green) and NeuN (red) revealed decreased fluorescence signal of MBP2 in BCCAO 3 m animals compared to sham 3 m control, and this effect was reversed by E2 treatment. NeuN staining in the hippocampal CA1 region did not appear to show any major difference between the groups, suggesting that there is no significant neuronal loss at 3 m BCCAO. Western blot analysis confirmed the decrease of MBP protein levels in the hippocampal CA1 region in 3 m BCCAO animals, and that E2 could reverse this effect (Fig. 3b & c). Furthermore, Western blot analysis revealed that compared to sham 3 m control, phospho-Tau (PHF1) protein levels increased slightly but significantly in the hippocampal CA1 level in BCCAO 3 m animals, and E2 treatment reversed this effect (Fig. 3b & d). Next, we performed electron microscopy (EM) to examine the ultrastructure alteration of myelin sheaths and oligodendrocytes in hippocampal CA1 region. As shown in Fig. 3e, EM ultrastructural analysis revealed that axons (green star) in the hippocampal CA1 region of BCCAO 3 m and Placebo treatment groups had abnormal myelin sheaths displaying severe disorganization (yellow arrow) and balloon-type swelling (blue star), while normal axon (green stars) with normal or only slight conformational changes of the myelin sheaths were observed in age-matched control and E2-treated animals. Additionally electron micrographs of oligodendrocytes from BCCAO 3 m animals showed dilatation of rough endoplasmic reticulum (blue arrow), swollen mitochondria with broken cristae (red star), while oligodendrocytes from sham 3 m and E2-treatment animals displayed normal morphology and intact mitochondria (green star). Quantification of the EM results confirmed that Pla 3 m markedly increased the percentage of damaged/total axons and swollen/total mitochondria in the hippocampal CA1 region, and that E2 treatment significantly attenuated these effects (Sup. Fig. 3). These results suggest that axonal damage occurs much earlier than neuronal loss during BCCAO, and that E2 can prevent these detrimental axonal changes.

### 17β-Estradiol prevents the loss of hippocampal dendritic spines induced by BCCAO 3 months

**Figure 4:**
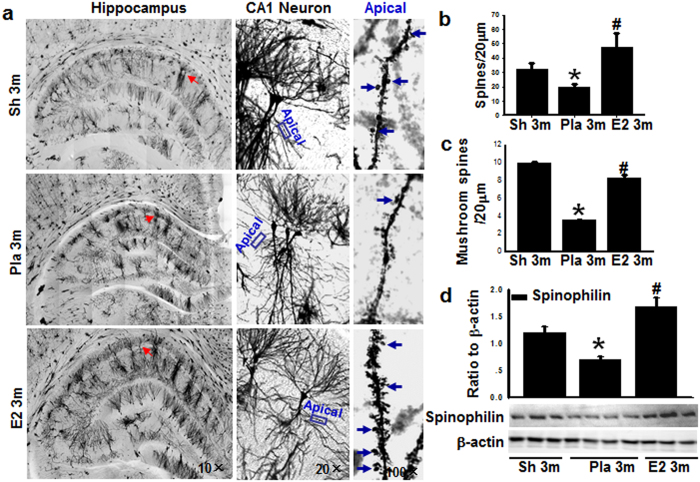
Micrograph of Golgi staining shows apical dendrites from hippocampal CA1 pyramidal neurons. **(a)** The left panel shows whole hippocampus (magnification 10×). The middle panel shows higher magnification of a pyramidal neuron from left panel marked by red arrow (20×). The right panel shows apical dendrites that are magnification of the boxes in the middle panel (100×). Blue arrows indicate mushroom spines. **(b)** Total number of spine per 20 μm apical dendrites. **(c)** Mushroom-like spine per 20 μm apical dendrites. **(d)** Western blot analysis for the spine marker spinophilin, and β-actin. Quantitative analysis for spinophilin shown as mean ± SE (n = 4–5 in each group). Data were expressed as ratio to β-actin. *P < 0.05 vs. sham 3 m control, ^#^*P* < 0.05 vs. Pla 3 m group.

Next we quantified the synaptic connections by counting total spines and mushroom-like spines using Golgi staining in the dendritic region of the hippocampal CA1 region. Because E2 treatment has been reported to increase spine density on CA1 neurons in ovariectomized female rats^33^, we first examined the effect of E2 on dendritic spine formation of CA1 neurons at 3 m in male rats that did not undergo BCCAO. Golgi staining analysis indicated that E2-treatment did not change total spine density of the hippocampal CA1 pyramidal neurons and behavioral outcome as compared to age-matched sham animals (sup. Fig. 4). Therefore, we next examined the ability of E2 to regulate spine density in the hippocampal CA1 region in animals that underwent 3 m BCCAO. As shown in Fig. 4a–c, BCCAO 3 m significantly reduced the density of total spines and the number of mushroom-like spines in the hippocampal CA1 region, as compared to sham 3 m controls, and this effect was reversed by E2. A similar enhancing effect of E2 on total spines and the number of mushroom-like spines in the hippocampal CA1 region was also noted in female rats (Sup. Fig. 5) Furthermore, Western blot analysis (Fig. 4d) showed that expression of spinophilin, a spine marker protein, was significantly attenuated in the male rat hippocampal CA1 region of the BCCAO 3 m group, as compared to the sham 3 m controls, and E2 prevented this effect. To further confirm the spine data in Fig. 4b and spinophilin data in Fig. 4d, a second experiment for Golgi staining and Western blot analysis was performed and almost identical results were obtained (Sup. Fig. 6).

### 17β-estradiol increases synaptic marker proteins in hippocampal CA1 pyramidal neurons after BCCAO 3 months

**Figure 5:**
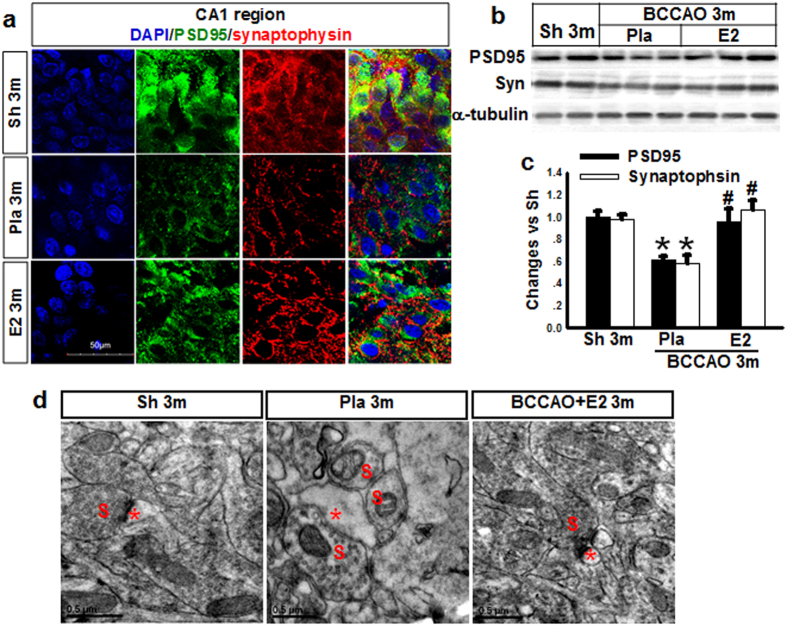
Effects of E2 treatment on synaptic markers (PDS95, Synaptophysin) and ultrastructure of synapses of hippocampal CA1 neurons at 3 m after BCCAO. (**a**) Immunofluorescent staining for DAPI (Blue), PSD95 (Green) and Synaptophysin (Red) in hippocampal CA1 region for various groups (Sham, Sh; Placebo, Pla; Estradiol, E2) n = 5, Scale bars = 50 μm and magnification 63×. (**b,c**) Western blot analysis for PSD95 and Synaptophysin. *P < 0.05 vs. sham 3 m group; ^#^*P* < 0.05 vs. Pla 3 m group. n = 5–6; (**d**) Ultrastructure of synapses of hippocampal CA1 neurons (S: Presynaptic; Red star: Postsynaptic).

To further confirm the synaptic regulatory effects of E2, we examined whether E2-treatment affected protein expression of synaptic marker proteins that are important for synaptic function and strengthening. In these studies, we examined expression in the hippocampal CA1 region of a presynaptic marker (synaptophysin) and the postsynaptic marker (PSD95) using double-immunofluorescent staining and Western blot analysis. As shown in Fig. 5a, BCCAO 3 m animals showed a marked decrease in the number of synaptophysin (red)- and PSD95 (green)-immunoreactive puncta in the CA1 region as compared to the 3 m controls. Furthermore, E2-treated animals showed marked preservation of synaptophysin- and PSD95- immunoreactive puncta in the hippocampal CA1 region, as compared with the BCCAO 3 m group. To further confirm these results, we utilized Western blot analysis, which confirmed the down-regulation of synaptophysin and PSD95 protein levels in the hippocampal CA1 region of BCCAO3m animals, and that E2-treated animals showed preservation of synaptophysin and PSD95 expression (Fig. 5b & c). Finally, we also performed ultrastructure analysis of excitatory synapses in hippocampal CA1 pyramidal neurons using EM. As shown in Fig. 5d, presynaptic and postsynaptic membranes were intact in the three groups, however in agreement with Western blot and immunofluorescent analysis, BCCAO 3 m significantly reduced the PSD thickness compared to sham 3 m, an effect that was reversed by E2-treatment.

### Long-lasting effects of 17β-estradiol on neuronal survival and learning and memory at 6 months after BCCAO

**Figure 6:**
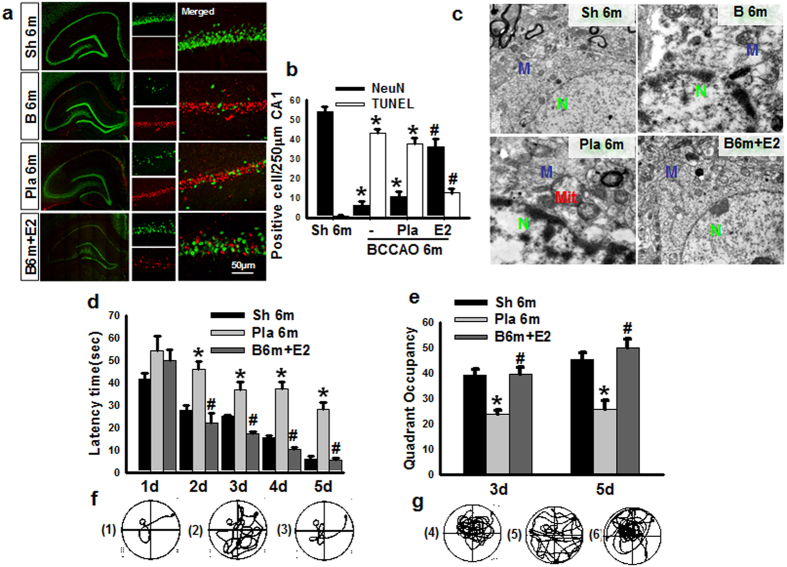
Administration E2 for 3 m decreases CA1 neuronal apoptosis and cognitive impairment after BCCAO 6 m. **(a)** Representative photographs for NeuN (Green) and TUNEL (Red) staining; **(b)** Quantify for NeuN- or TUNEL-positive cells in per 250 μm length of the medial CA1 pyramidal cell layer. n = 4–5, Scale bar = 50 μm and magnification 40×; *P < 0.05 vs. sham 6 m group, ^#^*P* < 0.05 vs. Pla 6 m group; **(c)** EM results show ultrastructure of CA1 neuron. N: nucleus; Mit: mitochondria; M: membrane. Latency trail **(d)** and Probe trail **(e)** in Morris water maze. *P < 0.05 vs. sham 6 m on the same day; ^#^*P* < 0.05 vs. Pla 6 m group on the same day. n = 6. Representative tracks for Latency trail **(f)** (a-sham 6 m, b-Pla 6 m, c- E2 6 m) and Probe trail **(G)** (d-sham 6 m, e-Pla 6 m, f- E2 6 m).

In the final set of experiments, we sought to determine whether animals treated with E2 during the first 3 m of BCCAO would still have beneficial effects on neuronal survival and cognitive function when examined at 6 months (6 m) after BCCAO. To examine neuronal survival and apoptosis in the hippocampal CA1 region, we used immunofluorescence staining for the neuronal marker, NeuN (green) and TUNEL analysis (red), respectively. Representative photomicrographs from all groups are displayed in Fig. 6a and the quantitative analysis is shown in Fig. 6b. The results demonstrate that BCCAO 6 m caused a significant decrease in neuronal survival (number of NeuN + cells) and an increase in apoptotic cells (number of TUNEL + cells) in the hippocampal CA1 region, as compared to sham controls (Fig. 6a & b). E2 treatment during only the first 3 months of BCCAO showed lasting beneficial effects at 6 m BCCAO, as evidenced by enhanced neuronal survival and reduced apoptosis in the hippocampal CA1 region, as compared to the BCCAO 6 m placebo control (Fig. 6a & b). Additionally, as shown in Fig. 6c, ultrastructure analysis of CA1 pyramidal neuron showed an irregular contour and an altered pattern of nuclear chromatin distribution in BCCAO 6 m and Pla 6 m groups, as well as swollen mitochondria with broken cristae, and dilated cisternae of rough endoplasmic reticulum, all signs of neuronal damage. In contrast, hippocampal CA1 neurons in E2-treated animals showed generally normal morphological appearance for neuronal nuclei and cytopasmic organelles. We next examined the ability of E2 treatment for the first 3 m of BCCAO to preserve cognitive function at 6 m BCCAO. Cognitive function in animals from all groups was examined using the Morris Water Maze (MWM) test. Latency trials were carried out from days 1 to day 5 to examine spatial learning ability (Fig. 6d), while probe trials were carried out on days 3 and 5 to examine spatial memory (Fig. 6e). Representative tracings for the latency and probe trials are shown in Fig. 6f & g, respectively. As shown in Fig. 6d–g, 6m BCCAO animals displayed a significant longer time in latency to find the platform (Fig. 6d & f), and a significant shorter time spent in the platform quadrant (Fig. 6e & g), as compared to 6 m sham controls. Importantly, the animals with continuously E2-treatment for the first 3 m exhibited significant preservation of cognitive function at 6 m BCCAO, as evidenced by a significantly decreased latency to find the platform and a significantly increased time spent in the platform quadrant, compared to BCCAO vehicle controls (Fig. 6d–g).

### The effects of E2-treatment on biomarkers of dementia in the hippocampal CA1 region after BCCAO 6 months

**Figure 7:**
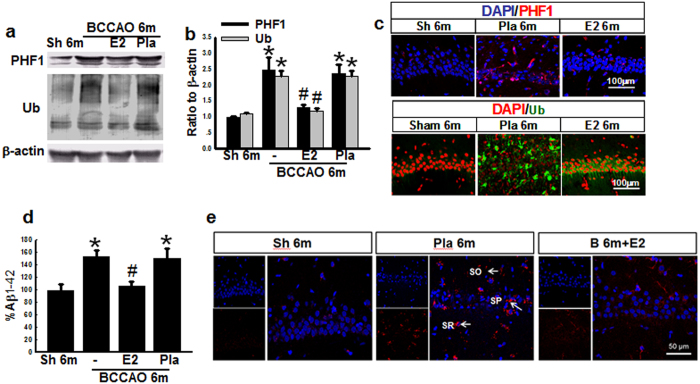
E2-treatment for 3 m decreases the protein expression of dementia biomarkers in hippocampal CA1 region 6 m after BCCAO. Hippocampal samples from sham (Sh) 6 m, BCCAO 6 m, Placebo (Pla) 6 m and Estradiol (E2)-treated groups for 3 m were subjected to Western blot analysis for PHF1 and total Ub **(a,b)**. **(c)** Representative photographs of immunofluorescent staining for PHF1 (Red) or total Ub (Green), with the nuclear stain, DAPI. **(d)** ELISA analysis for Aβ1-42, and **(e)** Immunofluorescent staining for Aβ1-42 (Red) and DAPI (Blue), indicating that Aβ1-42 is prominently deposited in hippocampal stratum radiatum (SR), stratum pyramidale (SP) and stratum oriens (SO) cell layers, and markedly decreased by E2 treatment. *P < 0.05 vs. sham 6 m group, ^#^*P* < 0.05 vs Pla 6 m group. n = 5-6. Scale bar: 50 μm and magnification: 40×.

Since AD and VD have some similarities in pathogenesis^30^,^34^, we next examined for changes in dementia-related biomarkers for neurofibrillary tangles (NFTs) and amyloid deposition. Hippocampal CA1 region protein samples were subjected to Western blot analysis for PHF1, which recognizes phosphorylated Tau and is a general marker for neurofibrillary tangles (NFTs), and total ubiquitination (Ub), an indirect marker for amyloid deposition in AD. As shown in Fig. 7a & b, PHF1 and Ub protein levels were markedly elevated in both BCCAO 6 m and Pla-BCCAO 6 m groups compared to age-matched sham controls, while continuous administration of E2 for the first 3 m significantly reversed the elevation. To further confirm the results, we performed double immunofluorescent staining for PHF1 and Ub coupled with the nuclear stain DAPI. Representative photographs in Fig. 7c show that there is a robust increase in PHF1- and Ub-labeled cells in the Pla-BCCAO 6 m group, as compared to sham 6 m controls, and that continuous administration of E2 for the first 3 m significantly reversed this elevation. To determine whether BCCAO could result in elevations of Aβ, we utilized ELISA to measure Aβ1-42 levels in hippocampal samples from the various groups. As expected, Aβ1-42 levels were markedly elevated in the BCCAO 6 m and Pla-BCCAO 6 m groups, as compared to the sham 6 m group, and continuous administration of E2 for the first 3 m significantly reversed this elevation (Fig. 7d). Furthermore, immunohistochemical analysis revealed that Aβ1-42 was prominently localized in the hippocampal stratum radiatum (SR), stratum pyramidale (SP), and stratum oriens (SO) cell layers in the Pla-BCCAO 6 m group, while both the 6 m sham control and E2-treatment groups showed very weak Aβ1-42 positive staining (Fig. 7e).

## Discussion

The results of the current study demonstrate for the first time that low dose E2 replacement in the BCCAO model of chronic cerebral hypoperfusion and VaD reverses BCCAO-induced reductions in cognitive impairment. An intriguing aspect of E2’s beneficial effect was that it was long lasting, as cognitive preservation by E2 was maintained at 6 m after BCAO even though E2 was only administered during the first 3 months of BCCAO. This finding suggests that early events in the pathogenesis of BCCAO, such as axonal and mitochondria damage and dendritic spine loss, have a critical role in induction of the later hippocampal neuronal cell death and long-term cognitive impairment. Furthermore, E2 suppression of these early pathogenic events can lead to long-term neurological benefit. In agreement with our BCCAO results, a number of other groups have also reported that BCCAO causes early white matter and axonal damage, as well as hippocampal synaptic plasticity defects that contribute to long-term cognitive deficits^4^,^35^,^36^.

There is growing recognition that reduction of white matter damage (in addition to reducing gray matter damage) is important to developing an effective translational agent in neurodegenerative disorders. Our finding that E2 can preserve morphology of myelin and oligodendrocytes and reduce markers of axonal damage in the BCCAO model may be a key factor in its neuroprotective and cognitive preservation effects observed at 6 m BCCAO. In addition, the ability of E2 to enhance pre- and post-synaptic proteins and prevent loss of hippocampal dendritic spines may be especially important for its beneficial cognitive effects, as dendritic spines have been shown to mediate excitatory neurotransmission and to be storage sites for synaptic strength^37^,^38^. Furthermore, loss of hippocampal dendritic spines has been shown to be strongly associated with cognitive impairment in a variety of neurodegenerative disorders, and prevention of dendritic spine loss correlates well with improved cognitive function^39^,^40^,^41^. The signaling mechanism underlying E2 effects upon dendritic spines after BCCAO remains unclear, however all three estrogen receptor types (ER-α, ER-β and GPER1) have previously been implicated to contribute to the synaptic plasticity effects of E2 in various regions of the brain^42^,^43^,^44^. Furthermore, we, and others previously reported that rapid kinase signaling involving ERK and Akt is critical for E2 enhancement of spinogenesis and synaptogenesis in cultured neurons^45^,^46^, raising the possibility that similar rapid kinase signaling may be involved in the enhancement of dendritic spines by E2 observed in the BCCAO model. Furthermore, ERK and Akt are well known pro-survival factors previously implicated in E2 neuroprotective effects following global cerebral ischemia^19^,^47^,^48^,^49^, and thus could also potentially mediate the anti-apoptotic, neuroprotective effects we noted for E2 at 6 m BCCAO.

Our study also demonstrated that markers for neurofibrillary tangles (PHF1), and amyloid deposition in AD (total Ub) were markedly elevated in the hippocampus of 6 m BCCAO animals. ELISA results further confirmed a robust elevation of Aβ1-42 levels in the hippocampus of 6 m BCCAO animals, as compared to sham controls. These findings are consistent with previous reports of Aβ1-42 elevation after BCCAO, and may be explained by a previous report of increased BACE1 expression, the initiating enzyme in Aβ generation^50^. In our study, low dose E2 treatment was able to strongly decrease the BCCAO-induced elevations of PHF1, Ub and Aβ1-42 in the hippocampus. The ability of E2 to decrease elevation of these dementia markers after BCCAO correlated with E2 preservation of cognitive function in BCCAO animals, suggesting a potential causative relation. Indeed, previous work has shown that the level of BACE1 and Aβ1-42 is *positively* correlated with cognitive impairment in BCCAO^50^.

It should be mentioned that previous work by our group has shown that E2 can also decrease vascular damage after chronic BCCAO. For instance, E2 increased VEGF levels, reduced hippocampal CA1 region vascular dilation, edema and leakage, endothelial cell damage, and increased VEGF protein expression^51^. Thus, in addition to the beneficial effects demonstrated in this study upon white and gray matter, spine density, dementia proteins, and cognitive function, E2 can also protect the vasculature in the hippocampus. Vascular and white matter damage are hallmarks of VaD, and the ability of E2 to attenuate damage to the vascular system, as well as white and gray matter raises the possibility that E2 may have benefit in VaD.

An important point in our study is that we observed significant beneficial effects using low doses of E2. From a translational standpoint this could be advantageous, as low doses of E2 may be less prone to cause potential negative side effects. However, it still may be best to consider the use of non-E2 analogues for translational studies, such as SERMs or other agents that retain beneficial neurological E2 actions, but lack E2’s potential detrimental tropic effects upon the uterus and breast. Along these lines, we recently demonstrated that the SERM, raloxifene, could enhance synaptic plasticity and neurogenesis in the brain after cerebral ischemia^25^. Raloxifene has been demonstrated to have beneficial effects upon bone like E2, but lacks E2 uterotrophic effect, and acts as an antagonist in the breast^52^. Likewise, genistein, a phytoestrogen that also lacks uterotrophic activity^53^, has been shown to be neuroprotective and to enhance cognitive function in acute focal and global cerebral ischemia animal models^54^,^55^. Finally, recent work suggests that it may be possible to utilize GPER1 ligands as a translational alternative to E2, as several groups including our own have demonstrated that the GPER1 agonist G1 enhances neuronal survival both *in vitro* and in *in vivo* in models of brain injury^47^,^56^,^57^,^58^. Additional work has shown that G1 also exerts vascular protective and anti-inflammatory effects *in vivo*, while lacking an uterotrophic effect^59^. Based on all of the above, preclinical studies to examine the neuroprotective and cognitive preserving ability of each of these non-E2 analogues in the BCCAO chronic hypoperfusion model are needed.

**Figure 8:**
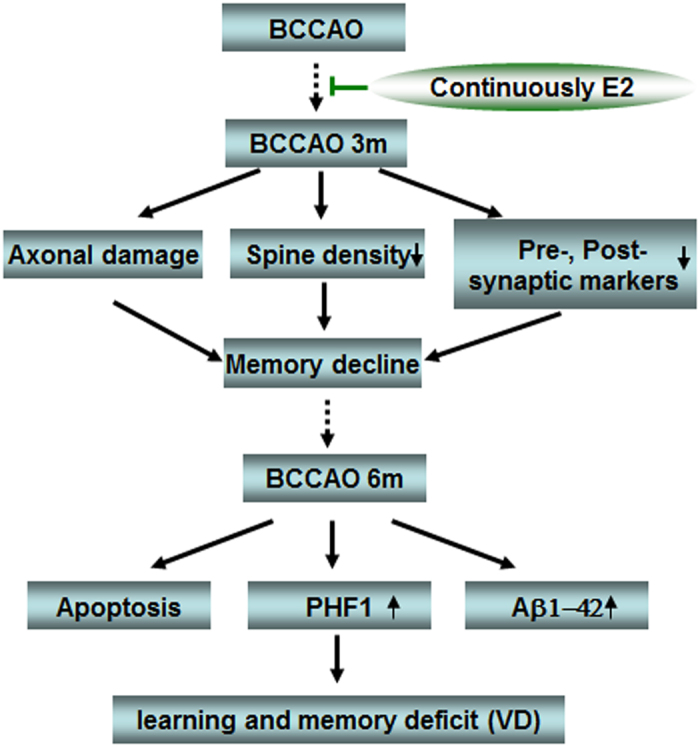
Summary diagram of the proposed mechanisms underlying progressive lesions in vascular dementia and the benificial effects of E2 administration during the first 3 months of BCCAO. The rats underwent BCCAO 3 m exhibit memory decline, which is caused by axonal damage, reduction in hippocampal spine density and synaptic proteins. E2 administration for 3 m reduces these key pathogenic events, and further to underlie the later beneficial effects on preventing apoptosis and dementia-related protein expression, as well as preservation of cognitive function observed at 6 months after BCCAO in E2-treated animals.

Based on the results of our study, Fig. 8 provides a hypothetical mechanism depicting the key pathogenic events that occur following BCCAO that contribute to long-term cognitive impairment, and illustrate the beneficial effects of E2 administered during the first 3 months of BCCAO. E2 treatment during the first 3 months of BCCAO attenuated early pathogenic events such as axonal damage, reduction in hippocampal spine density and synaptic proteins. E2 ability to reduce these key pathogenic events is believed to underlie the later decrease of apoptosis and dementia-related proteins and preservation of cognitive function observed at 6 months after BCCAO in E2-treated animals. Taken as a whole, the current findings raise the possibility that low does E2 or other non-E2 analogues may be an effective therapy to preserve neurological health and cognitive function following chronic cerebral hypoperfusion and VaD.

## Materials and Methods

### Antibodies and Reagents

MAP2 (A-4, sc-74421), MBP2 (10458-1-AP, Proteintech, A-3, sc-376995), β-actin (sc-81178) and Ubiquitin (Ub) (P4D1, sc-8017) were from Santa Cruz Biotechnology, Inc. Postsynaptic density 95 (PSD95) (ab18258), and synaptophysin (ab8049) were from Abcam. NeuN (MAB377) was from Millipore Biotechnology. Anti-PHF1 primary antibody is a gift kindly from Dr. Peter Davies, Albert Einstein College of Medicine. Alexa-conjugated secondary antibodies were from Molecular Probes/Invitrogen (Carlsbad, CA). TUNEL (Terminal deoxynucleotidyl transferase-mediated Dutp nick end labeling) was carried out using the ‘*In Situ*” Cell Death Detection Kit, Fluorescein’ kit (Cat #11684795910, Roche Molecular Biochemicals. Golgistaining kit (FD Rapid GolgiStain ^TM^ Kit) was from FD Neurotechnologies. INC. Amyloid beta1–42 ELISA kit (KMB3441) was from Thermo Fisher Scientific Inc. Polyvinylidene difluoride (PVDF) membranes with pore size of 0.45 or 0.2 μm were from Millipore Biotechnology (USA). BCIP (5-bromo-4-chloro-3-indolyl-phosphate) and NBT (nitro blue tetrazolium) were from Promega (Madison, WI). All the other chemicals were from Sigma-Aldrich (St. Louis, MO) unless indicated otherwise.

### Animal model of vascular dementia

Adult male Sprague Dawley (SD) rats (weighing 250–300 *g*, Beijing HFK Bioscience Co. Beijing, China) were used through the research. The animals were housed in cages with *ad libitum* food and water and were maintained on a 12 h light-12 h dark cycle in a temperature-controlled room (23 ± 1 °C). All procedures used were approved by Institutional Animal Care and Use Committee of North China University of Science and Technology and were conducted in accordance with the guidelines for animal research of National Natural Science Foundation of China. Bilateral common carotid artery occlusion (BCCAO) was carried out in the rats according to Lana *et al*.^60^. Briefly, animals were anesthetized using 10% choral hydrate (700 mg/kg, *ip*) and the right common carotid artery of the rats was bluntly separated and permanently occluded with a silk suture, and 7d later the same procedure was performed to occlude the left common carotid artery to produce BCCAO. Placebo (20% cyclodextrin) or 17β-estradiol (E2) minipumps (Alzet osmotic minipumps; model 2006, 6 weeks release, 0.15 μl/h, 200 μl; Durect Corporation) were subcutaneously implanted in the upper mid-back region after BBCAO and continued until the end of each experiment. The dose of E2 was 0.05 μg/h release, which produces serum E2 levels of physiological low diestrus levels of E2 (10–15 pg/ml) in female rats^61^. Rectal temperature was maintained at 36.5–37.5 °C throughout the procedure using a heat pad. The animals in the sham group underwent identical procedures, except the common carotid arteries were simply exposed without occlusion.

### Tissue preparation and Western Blotting

Rats were killed under anesthesia at specified time-points, as detailed in the experiments. The hippocampal CA1 tissues were microdissected from both sides of the hippocampal fissure and immediately frozen in liquid nitrogen. The tissues were homogenized in ice-cold tissue protein extraction buffer consisting of (in mM) the following: 50 HEPES Ph 7.4, 150 NaCl, 1 β-glycerophosphate, 3 DTT, 2 Na_3_VO_3_, 1 EDTA, 1 EGTA, 1 NaF, 1 phenylmethylsulfonyl fluoride (PMSF), 1% Triton X-100 and Protease & Phosphatase Inhibitors Cocktail (#1861280, Thermo Scientific. USA). The homogenates were centrifuged at 15 000 *g* for 30 min at 4 °C and then the supernatants were collected and stored at 80 °C until use. An enhanced BCA protein assay kit (P0009, Beyotime Institute of Biotechnology, China) with bovine serum albumin (BSA) as the standard was used to determine the protein concentrations. Protein from each sample was heated at 100 °C for 5 min with loading buffer containing 0.125 M Tris-HCL (PH 6.8), 20% glycerol, 4% SDS, 10% mercaptoethanol and 0.002% bromphenol blue, then separated by sodium dodecyl sulfate-polyacrylamide gel electrophoresis (SDS-PAGE) of 4–20% gels (50 μg protein per lane). Then the proteins were transferred onto PVDF membranes using a wet transfer system at 300 mA for 60 min. Blotting membranes were incubated with 3% BSA, 0.2% Tween 20 in TBST for 1 h at room temperature and probed with corresponding primary antibodies at 4 °C overnight. After washing with TBST for 3 × 10 min, the PVDF membranes were probed with alkaline phosphatase conjugated IgG and developed with BCIP-NBT. The membranes were scanned and the intensities of the bands were quantified using ImageJ analysis software (version 1.30 v; Wayne Rasband, National Institutes of Health, Bethesda, MD). The band densities for the indicated proteins were corrected for variations in loading and normalized relative to β-actin. Normalized means were then expressed as fold changes of the corresponding value for sham operated animals. A mean ± SE was calculated from the data from all of the animals for graphical presentation and statistical comparison.

### Morris Water Maze (MWM)

Cognitive deficits in spatial learning and memory of the rats were evaluated with Morris water maze, as previously described with minor modification^62^,^63^. The latency period (learning phase) involved four trials per day for seven consecutive days in which the rats were placed in the water facing the wall in random one of the four quadrants (I, II, III and IV) so that all four quadrants are used once a day. For each trial, the rat is allowed to swim a maximum of 90 s to find the hidden platform. When successful, the rat is allowed a 20 s rest period on the platform. If unsuccessful within 90 s, the rat is given a score of 90 s and is then physically placed on the platform and allowed the 20 s, assuring that each rat has equal time to observe spatial cues after each trial. The latency time, representing the average of the four trails to reach the platform and swimming speed were recorded. On day 3 and day 7 (6 h after acquisition) probe trial was conducted in which the platform is removed from the pool to measure spatial bias for the previous platform location. During probe trial, each rat was placed in the pool from a same quadrant and allowed to swim for 90 s. The percentage of time spent in the previous target quadrant and the number of crossings over the previous platform location were recorded.

### Histological Analysis and Immunohistochemistry staining

Histological examination of the ischemic brains was carried out as our previously described^64^. The rats were deeply anesthetized with isoflurane and subjected to transcardial perfusion with 0.9% saline followed by 4% paraformaldehyde. Brains were isolated and further fixed in 4% paraformaldehyde overnight, followed by incubation in 30% sucrose until they sank and were embedded in OCT compound. Frozen sections (25 μm) each were cut in series, using cryotome in the coronal plane of the dorsal hippocampus (~2.5–4.5 mm posterior from the bregma). Double immunofluorescence staining for NeuN, neuronal marker, and TUNEL, DNA damage and apoptosis marker (TUNEL kit, lot #1639496, Life Technologies) were performed to investigate the neuroprotective effect of E2 following BCCAO. Briefly, the sections were washed using 0.1 M PBS for 30 min, permeabilized with 0.4% Triton X-100-PBS for 1 h, blocked in 10% donkey serum for 1 h, and then incubated in anti-NeuN antibody (1:800) overnight at 4 °C. After rinsing three times over 40 min with 0.1% Triton X-100-PBS, the sections were incubated with secondary antibodies (Alexa Fluor 488 nm donkey anti-mouse IgG) at room temperature for 1 h followed by washing for 4 × 10 min in 0.1% Triton X-100-PBS. The sections were incubated in terminal deoxynucleotidyl transferase (TdT) reaction buffer A for 10 min at 37 °C and then in TdT reaction mixture, including enzyme solution, for 1 h at 37 °C. After 5 min washing with distilled water, the sections were incubated in Click-It Plus TUNEL reaction mixture for 30 min at 37 °C, washed with 0.1% Triton 100-PBS over 20 min, and then mounted on slides covered with water-based mounting medium. The number of NeuN-positive or TUNEL-positive neurons in immunofluorescent staining, or survival cells in NeuN staining per 250 μm length of the medial CA1 pyramidal cell layer was bilaterally counted in five sections per animal and was averaged to provide the mean value. A mean ± SE was calculated from the data in each group and statistical analysis performed as described below.

For immunofluorescence staining, brain coronal sections were processed with same procedure until adding primary antibody. The following primary antibodies were used in different combinations: anti-NeuN (1:800) and anti-MAP2 (1:100) and anti-MBP2 (1:100); anti-PSD95 (1:500); anti-synaptophysin (1:500); PHF1 (1:1000); Ub (1:100). The sections were incubated in primary antibody for 48 h at 4 °C and were washed four times at room temperature, followed by incubation with appropriate fluorescent-labeled secondary antibodies (1:200) for 1 h at room temperature. If necessary, DAPI was incubated for counterstaining of the nucleus. All the confocal images were captured on a laser scanning confocal microscope (LSCM, Olympus FV1000) and Digital imaging software (FV10-ASW 1.5 Viewer).

### Transmission electron microscope (TEM)

Analysis of ultastructural morphology was performed using TEM as described previously by our group and others^65^,^66^. Briefly, the obtained brain tissue from the Stratum radiatum (100–200 μm from soma) and stratum pyramidale of hippocampal CA1 region were diced into proper blocks (1 mm^3^), and immediately fixed in 2.5% glutaraldehyde (phosphate buffer, Ph 7.2) overnight at 4 °C, and then in a mixed solution of 2.5% glutaraldehyde with 2.0% paraformaldehyde for 4 h at 4 °C. This was followed by three washes in 0.1 M PBS for 15 min each. The tissues were then post-fixed with 2% osmium tetroxide for 30 min, and then dehydrated in a series of graded ethanol solutions. Subsequently, ethanol was substituted with propylene oxide, and then embedded in Epon 812. Ultrathin sections (70 nm thickness) were mounted on 200-mesh copper grids. The copper grids were counterstained with uranyl acetate (30 min) and then lead citrate for 10 min. Finally, the copper grids were washed with PBS and distilled water, and the ultrastructure was observed by transmission electron microscope (H Hitachi-7650), micrographs were taken and analyzed carefully for fine structural changes.

### Golgi Staining and semiquantitative analysis of spine density

Golgi-Cox staining was performed using a kit following the protocol of the manufacturer (FD Rapid GolgiStain^TM^ Kit, FD NeuroTechnologies, Inc.) with minor alterations. In brief, the rats were deeply anesthetized with 10% chloral hydrate before killing and perfused transcardially with cold 1% paraformaldehyde in phosphate-buffer saline (PBS; Ph7.4) for 1 minute. The animal brains were removed from the skull as quickly as possible and sliced into blocks containing entire dorsal hippocampus (approximately 1.5–5.5 mm posterior from bregma). The tissue blocks were immersed in an impregnation solution (mixture of equal volumes solutions A and B, prepared at 24 h prior to use) at room temperature (RT) for 3 weeks with replace the impregnation solution on the next day. Next, the blocks were transferred into solution C at RT for 5d with changing the solution C on the next day. The blocks were cut into sections (200 μm) on a vibratome and then mounted on gelatin-coated microscope slides with a dropping of solution C immediately. After air-dry naturally at RT overnight and three 5-min washes with Milli-Q water, the sections were incubated in the working solution (a ratio 1:1:2 of solution D: E: Double distilled water) with gently shaking for 10 min. After three 5-min washes in Milli-Q water, the sections were dehydrated in increasing concentrations of ethanol (50%, 75%, 90% and 100% I, II, III 5 min each), followed by clearing in, 3 times 10-min xylene and coverslip with permount. During processing, the brain sections were protected from light whenever possible. A series of every 10^th^ section through the entire hippocampus block of each animal was used in semiquantitative analysis of spine density, resulting in 5 sections per group (n = 5 in each group) on average. Z-stack images (z-step 2 μm) of 5 neurons at least from each section (25 neurons in total, per group) were captured under a Zeiss Icore-510 upright confocal scanning laser microscope using an oil-immersion objective (63 × 1.5NA) and were three-dimensionally reconstructed by NeuroLucida Software (MicroBrightField), noting that only those neurons were selected in which both the soma and the entire dendritic tree was impregnated. Those primary and secondary apical dendrites (at least 20 from each cell) were selected for quantitative analysis of which at least a 10 μm segment was in the plane of focus. The lengths of these segments were measured and the spines counted and spine density values were expressed as number of spines/20 μm of dendrite. Furthermore spines with a head diameter of at least ~0.3 mm were deemed as mushroom spines. Sections from each animal were coded so that the individual who performed the quantitative analysis and spine quantification on the sections did so in a blind fashion.

### ELISA analysis for Aβ1-42 Levels

Aβ1-42 levels in hippocampal CA1samples (30 μl, 1 μg/μl) were measured using a commercially available Aβ1–42 ELISA Kit (KMB3441) (Thermo Fisher Scientific Inc.) according to the protocol provided by the manufacturer. The plates were read at 450 nm, and the data based on the standard curve were expressed as fold changes vs. age-matched control.

### Statistical Analysis

Statistical analysis was performed using either one-way or two-way analysis of variance (ANOVA), followed by Student-Newman-Keuls post-hoc tests to determine group differences. When only two groups were compared, a Student’s T test was used. Statistical significance was accepted at the 95% confidence level. Differences of *P* < 0.05 were considered significant. All values were expressed as the means ± SE.

### Ethical Approval

All procedures used in this study were approved by Institutional Animal Care and Use Committee of North China University of Science (Ref. 20120117) and Technology and were conducted in accordance with the guidelines for animal research of National Natural Science Foundation of China.

## Additional Information

**How to cite this article**: Zhu, Y. *et al*. Protective Effect of 17β-Estradiol Upon Hippocampal Spine Density and Cognitive Function in an Animal Model of Vascular Dementia. *Sci. Rep.* **7**, 42660; doi: 10.1038/srep42660 (2017).

**Publisher's note:** Springer Nature remains neutral with regard to jurisdictional claims in published maps and institutional affiliations.

## Supplementary Material

Supplementary Information
