# Supplementary material for: Protective Effect of 17β-Estradiol Upon Hippocampal Spine Density and Cognitive Function in an Animal Model of Vascular Dementia
Source: Sci Rep. 2017 Feb 16;7:42660. doi: 10.1038/srep42660 (PMC5311994; doi:10.1038/srep42660)
Supplement: Supplementary Information [file srep42660-s1.pdf]

Supplementary Information

Protective Effect of 17β-Estradiol Upon Hippocampal Spine Density and Cognitive Function in an Animal Model of Vascular Dementia

Ying Zhu[1a], Quanguang Zhang[2a], Wenli Zhang[1], Ning Li[1], Yongxin Dai[1], Jingyi Tu[1], Fang Yang[1], Darrell W. Brann[2]*, Ruimin Wang[1]*

[1]Neurobiology Institute of Medical Research Center, International Science & Technology Cooperation Base of Geriatric Medicine, North China University of Science and Technology, Tangshan 063000, China;

[2]Department of Neuroscience and Regenerative Medicine, Medical College of Georgia, Augusta University, Augusta 30912, USA;

***Correspondence to:** Ruimin Wang, Ph.D.
North China University of Science and Technology, Tangshan 063000, China,
Tel : 86-3725712, Fax : 86-3725495
Email: ruimin-wang@163.com

***Co-Correspondence to:** Darrell Brann, Ph.D.
Augusta University, Augusta 30912, USA
Tel: 10 706-721-7779
Email: dbrann@augusta.edu

[a] These authors contributed equally to this work.

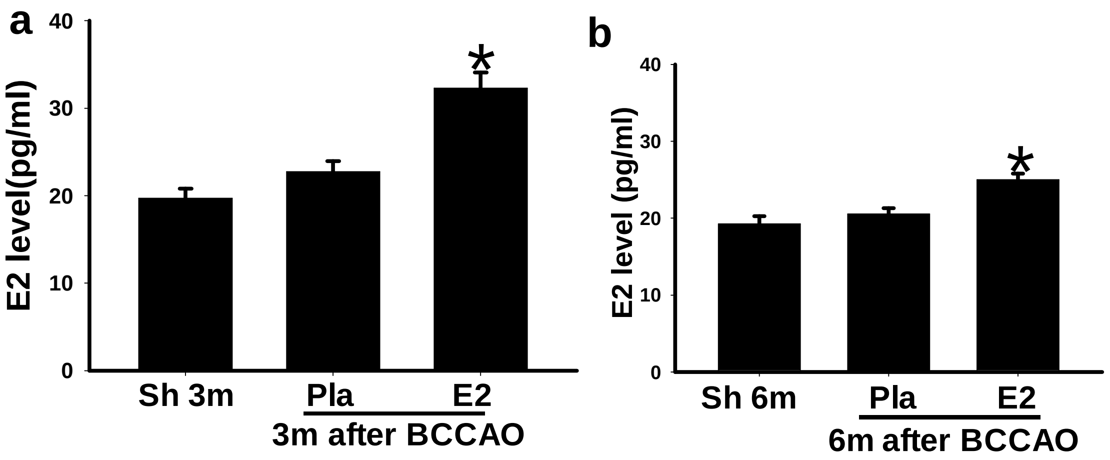

Sup. Figure 1. Circulating E2 levels at 3m (a) and 6m (b) animals. Serum samples from sham 3m and 6m, Pla 3m and 6m, E2 3m and 6m were subjected for ELISA analysis of 17β-estradiol. Data are expressed as mean±SE from 10 rats (3m) and 5 rats (6m). **P* <0.05 vs. the same time-point Pla group.

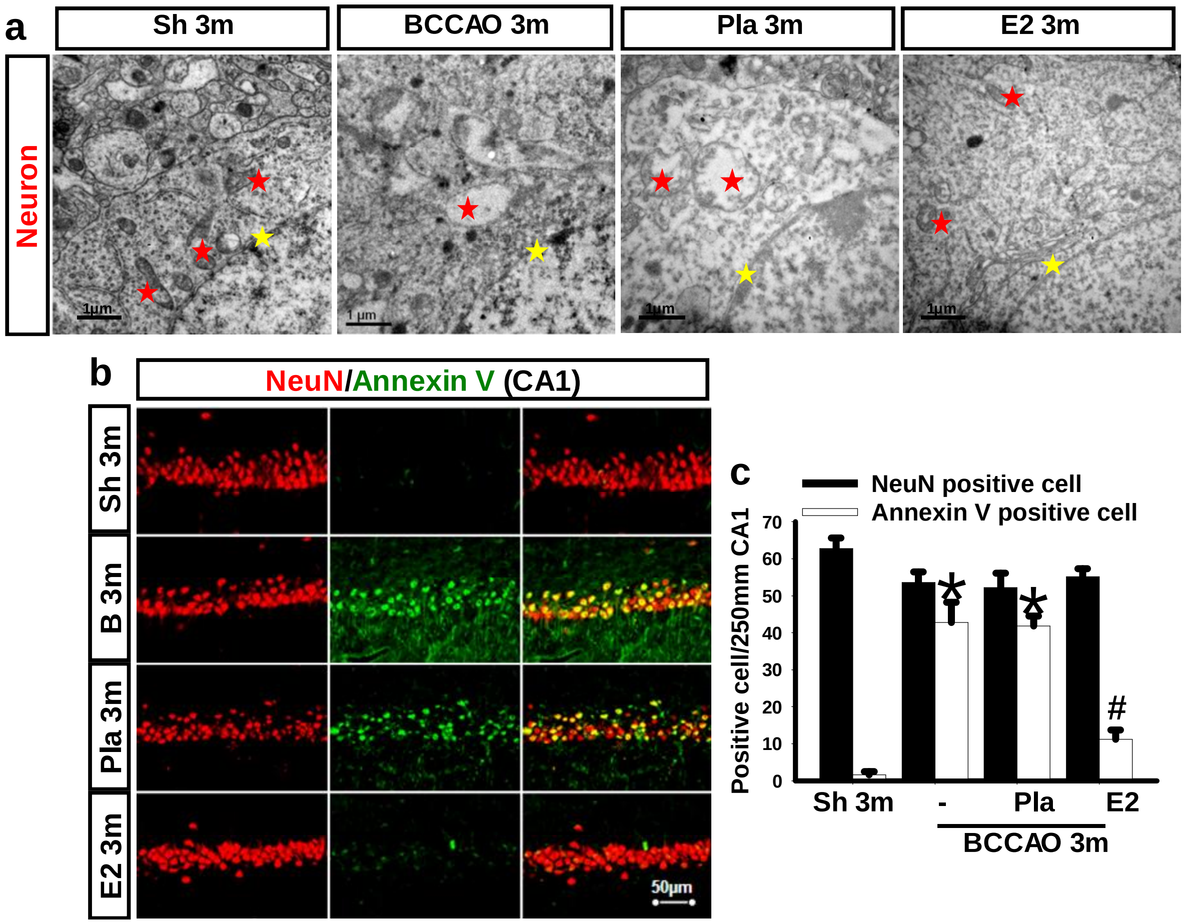

Sup. Figure 2. Effects of E2-treatment upon neuronal ultrastructure and early apoptotic marker Annexin V in hippocampal CA1 region at 3 months after BCCAO. (a) Ultrastructure of neuron (Yellow star: nuclear membrane; Red star: mitochondria), showing strikingly enlarged mitochondria with broken cristae and a decrease in cytoplasmic organelles in BCCAO 3m and Pla 3m groups compared with the normal mitochondria shown in Sham 3m and E2-treatment groups. **(b)** Double-immunofluorescent staining for Annexin V (Green) and NeuN (Red). **(c)** Quantify for NeuN- or Annexin V-positive cell per 250 μm length of the medial CA1 pyramidal cell layer. n=5-6, Scale bars 50 μm and magnification 40×; $^{*}P<0.05$ vs. sham 3m group; $^{\#}P<0.05$ vs. Pla 3m group.

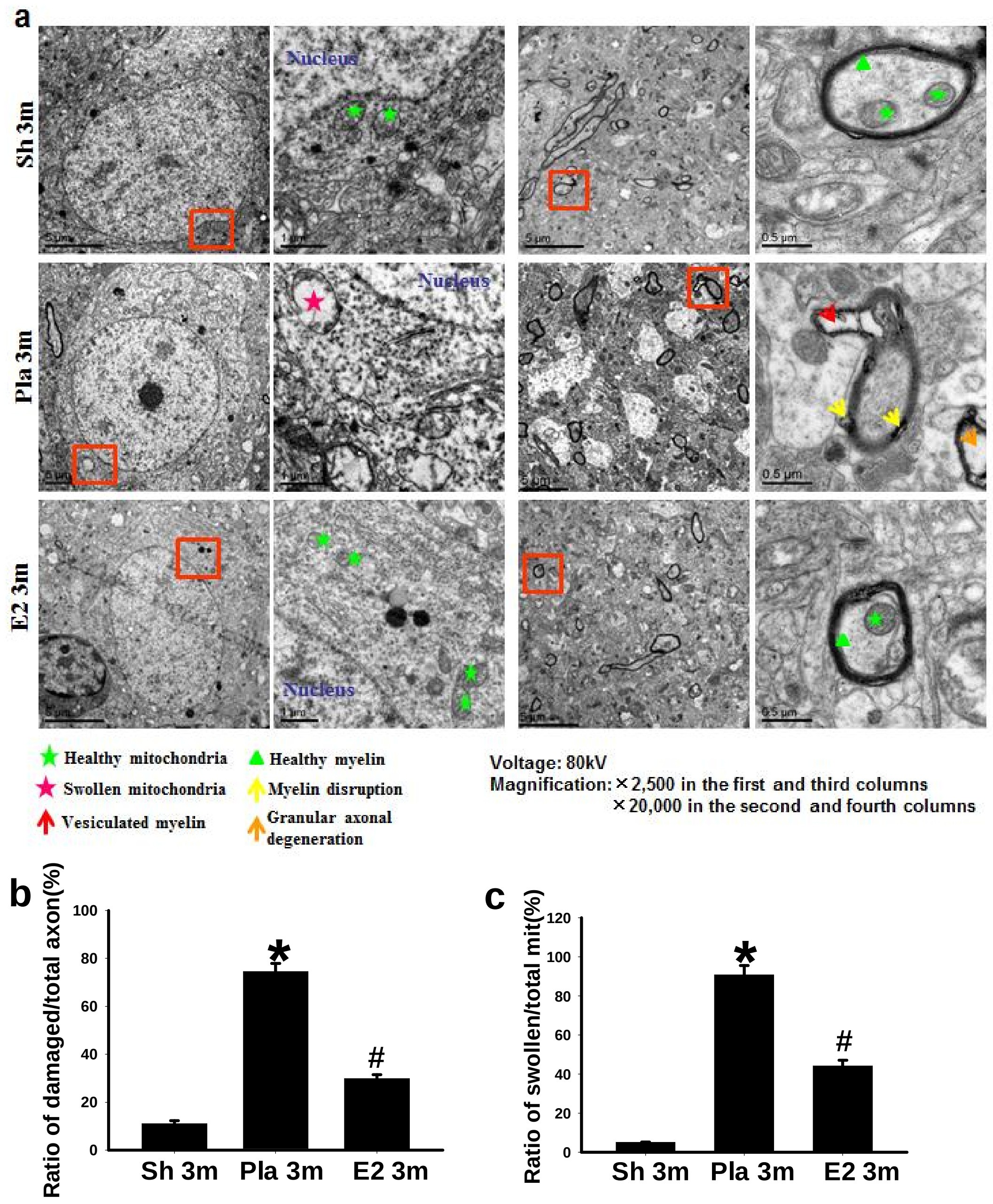

Sup. Figure 3. Quantification analysis of damaged axons and swollen mitochondria in hippocampal CA1 region. (a) Representative photomicrographs from indicated groups **(b)** and **(c)** Quantification of the percentage of damaged/total axons or swollen mitochondria respectively. $^{*}P$<0.05 vs sham, $^{\#}P$< 0.05 vs Pla 3m. The scale bar is indicated in the photograph. n=6-8

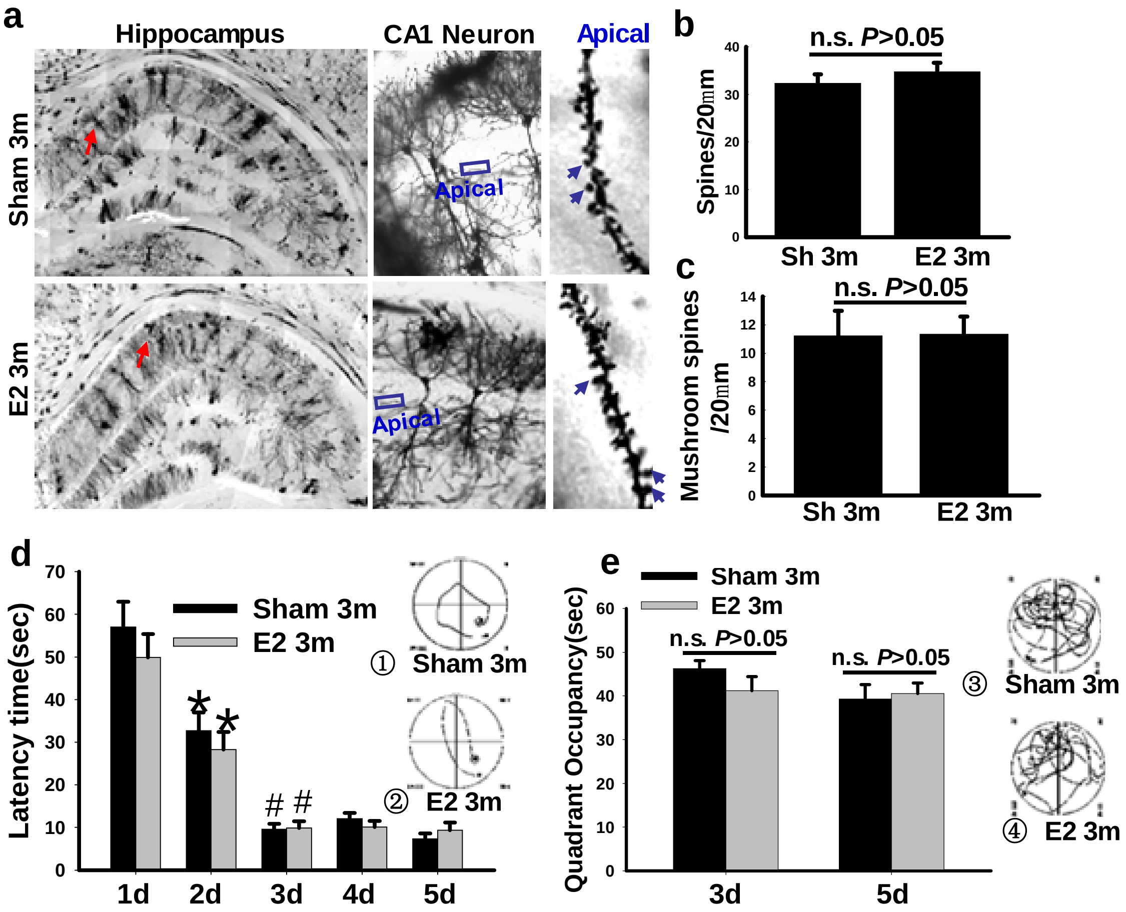

Sup. Figure 4. Golgi staining of the hippocampal CA1 area prepared from sham 3m or E2-treated for 3m. (a) The left panel shows whole hippocampus (magnification 10×), the middle panel shows higher magnification of a pyramidal neuron from left panel marked by red arrow (20×) and the right panel shows apical dendrites that are magnification of the boxes in the middle panel (100×). **(b)** Total number of spines per 20 µm apical dendrites. **(c)** Mushroom-like spines per 20µm apical dendrites. Blue arrows indicate mushroom spines. **(d)** Latency trial and **(e)** probe trail in MWZ. Representative traces of latency trail at day 5 (① sham, ②sham+E2) and probe trail (③sham, ④ sham+E2). $^*P<0.05$ vs the same group animals at day one, $^{\#}P < 0.05$ vs the same group animals at day two. n.s. means no significant change (P>0.05).

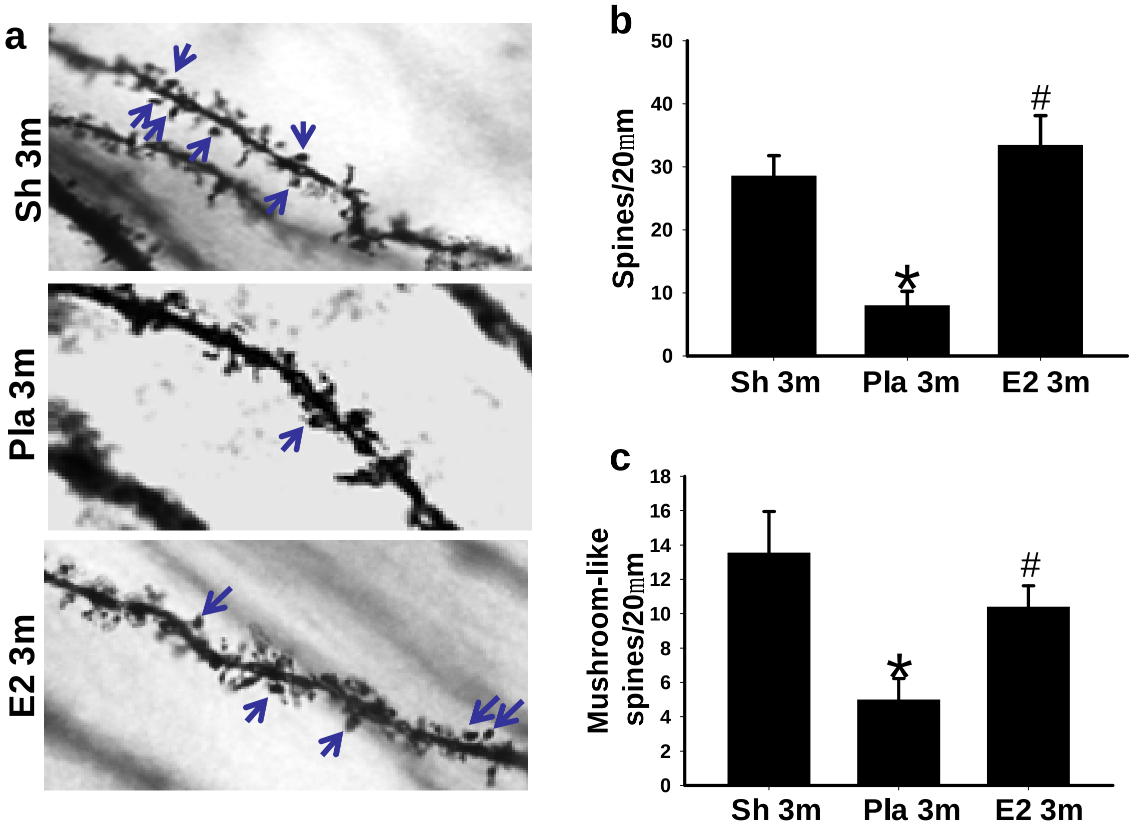

Sup. Figure 5. Golgi staining of the hippocampal CA1 area in OVX-female rats at 3m. (a) Representative photographs of apical dendrites in indicated groups (100×). **(b)** Total number of spines per 20 μm apical dendrites. **(c)** Mushroom-like spines per 20μm apical dendrites. Blue arrows indicate mushroom spines. n=6-8

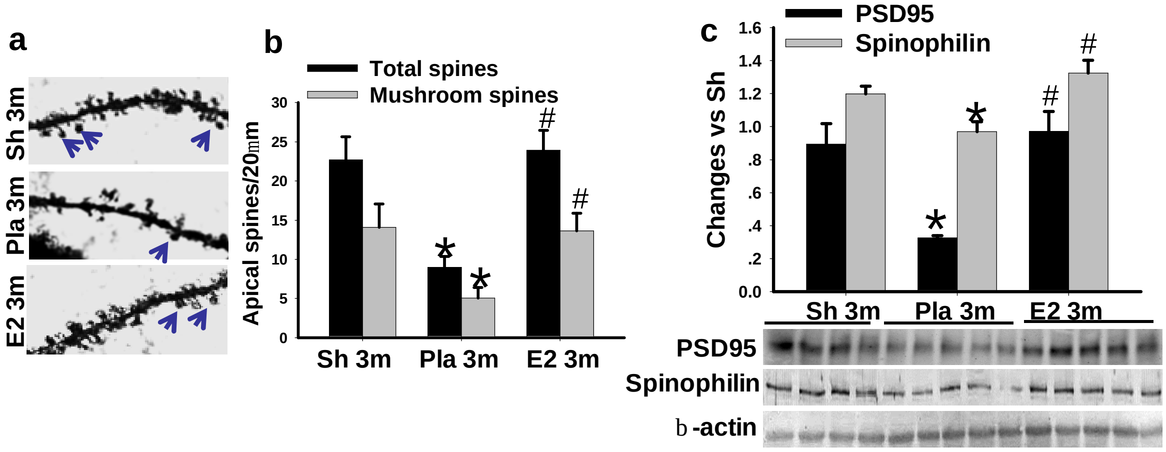

Sup. Figure 6. Effects of E2 treatment on apical spine, PDS95, Spinophilin of hippocampal CA1 neurons at 3m after BCCAO. (a) Representative photographs of apical dendrites in indicated groups (100×). **(b)** Total and mushroom-like spines per 20 μm apical dendrites. Blue arrows indicate mushroom spines. n=4-5. **(c)** Western blot analysis for PSD95 and Spinophilin, n=4-5. **P*<0.05 vs. sham 3m group; $^{\#}P$<0.05 vs. Pla 3m group.

Materials and Methods

The antibody of Annexin V (ab54775) was from Abcam.

The detailed protocols for immunofluorescence staining [Annexin V (1:500) and NeuN (1:800)], TEM and Golgi staining, as well as statistical analysis have been described in the main text.

Surgery in female animals and 17b-estradiol treatment

Adult Sprague Dawley female rats (body weight 250–300 g) were obtained from Beijing HFK Animal Center (SCXK(JING)2006-0009) and used in the study. BCCAO and 17β-estrodial (E2) administration were carried out at 1 week after bilaterally ovariectomized (OVX) under anesthesia using the same protocol as described with male animals. The rats were scarified at 3m after BCCAO.

Quantification of 17b-estradiol levels

E2 levels in serum samples from 3m and 6m animals were measured using a sensitive ELISA method as described previously by our group (Zhang et al., 2014). The results of the ELISA revealed that E2-treated male animals had a significant increase in circulating E2 levels at 3 and 6 months as compared to controls (**Sup. Figure 1**). The 17β-Estradiol ELISA (ADI-900-174, EnzoLife Sciences, Farmingdale, NY) used in the study has a detection range of 15.6–1000 pg/ml. Briefly, 100 μl of serum sample was added to the bottom of the appropriate wells followed by the addition of 50 μl 17β-estradiol conjugated to alkaline phosphatase and 50 μl sheep polyclonal antibody to 17β-estradiol. The plate was then incubated at room temperature with shaking (~500 rpm) for 2 h. After 3 washes with 400 μl

of wash buffer, 200 μl of the pNpp substrate solution was added into each well and incubated for 1 h at room temperature without shaking. Stop solution (50 μl) was added afterwards and the optical density was read at 405 nm. 17β-estradiol levels in the samples were determined based on an established standard and expressed as pg/ml.

Quantification of damaged axons and mitochondria in hippocampal CA1 region

Swollen and total mitochondria were counted in the soma of pyramidal neurons, while axons with myelin alterations were counted in the stratum radiatum of CA1 region. Briefly, 10 different fields from specimens (n=6-8) of each experimental group were selected, and myelinated axons and mitochondria were observed randomly. EM images were photographed at lower (×2,500) to higher (×20,000) magnifications and semi-quantitative analysis was carried out by observation of the ultrastructural features of approximately 300 randomly selected myelinated axons and mitochondria per group. Damaged axons were identified as those with splitting of the myelin sheath, focal damage in myelin sheath or vesiculation, and granular degeneration of axons. Data was expressed as percentage of damaged axons or swollen mitochondria divided by total myelinated axons or mitochondria.
